# A case of endovascular treatment for intestinal ischemia due to acute superior mesenteric artery embolism after TEVAR

**DOI:** 10.1093/jscr/rjz185

**Published:** 2019-06-14

**Authors:** Masahiro Tsutsui, Norihumi Ootani

**Affiliations:** Department of Cardiac Surgery, Sapporo Teishinkai Hospital, Sapporo, Japan

## Abstract

Recently, the use of thoracic endovascular aortic repair (TEVAR) for the treatment of aortic aneurysm is increasing. Therefore, complications associated with TEVAR are expected to increase. Here, we present a case of thrombus aspiration and percutaneous transluminal angioplasty (PTA) for superior mesenteric artery (SMA) embolism after TEVAR. A 69-year-old male patient with an aortic arch aneurysm received TEVAR. On the fourth post-operative day, he suddenly complained of strong abdominal pain. Since enhanced computed tomography (CT) showed SMA embolism, we urgently performed thrombus aspiration and PTA in two consecutive days. After these operations, almost all revascularization was achieved, although a partial occlusion remained. As a result, his symptoms improved, and no recurrence occurred. With the increased use of TEVAR, associated embolism may also increase. The combination of thrombus aspiration and PTA could be one effective option for such cases.

## INTRODUCTION

The number of thoracic endovascular aortic repair (TEVAR) performed for aortic aneurysm is increasing every year. Therefore, TEVAR-associated complications also are likely to increase. Embolism is one of the complications of TEVAR. Among them, superior mesenteric artery (SMA) embolism is a rare but lethal condition, which is necessary to diagnose and treat rapidly. Here, we present a case treated with thrombus aspiration and percutaneous transluminal angioplasty (PTA) for SMA embolism after TEVAR.

## CASE REPORT

**Figure 1:**
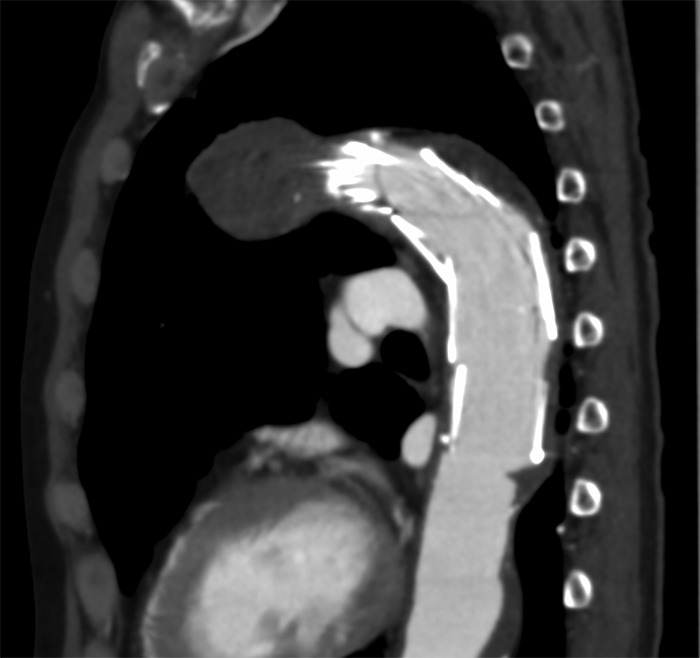
The distal side of the stent graft was placed to stick the thrombus of the aorta.

A 69-year-old male patient was admitted to our department with a diagnosis of three aortic aneurysms. His history included severe chronic obstructive pulmonary disease and hypertension. The aneurysms consisted of aortic arch saccular aneurysm with a protruding diameter of 25 mm, descending aortic aneurysm with a protruding diameter of 18 mm, and abdominal aortic aneurysm with a diameter of 36 mm. Of these, saccular aneurysm of the aortic arch required prompt treatment. We performed TEVAR using Najuta (Kawasumi, Tokyo, Japan) for this specific aneurysm and the operation was successful. After the operation, the patient complained of mild abdominal pain but on examination, there was no abdominal tenderness and the blood test showed no acidosis or abnormality. On the fourth post-operative day, patient suddenly complained of strong abdominal pain. Enhanced CT showed SMA embolism about 52 mm in length from the ileocolic artery bifurcation. As the distal side of the stent graft was placed to stick the thrombus of the aorta, it was thought that the embolus may have been liberated from this point (Fig. 1). We diagnosed SMA embolism and emergently performed revascularization with intervention radiology (IVR).

**Figure 2:**
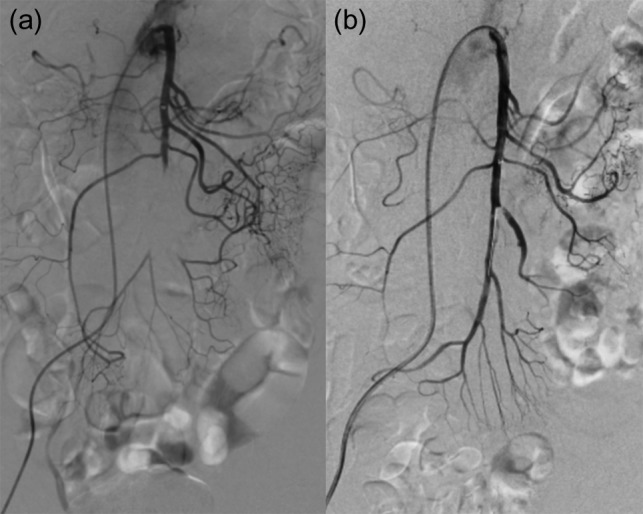
(a) pre IVR contrast injection (b) post IVR contrast injection.

The operation initiated with the patient in the supine position. A 6-French (Fr) guiding sheath was inserted into the right femoral artery. Subsequently, a 4-Fr shepherd hook catheter was placed in the SMA, and then the 0.035-inch guidewire was left in the SMA, and the shepherd hook catheter was changed to a 4-Fr straight catheter. Using this catheter as a foundation, the guiding sheath was placed into the SMA. The contrast injection as well as the CT image showed that the SMA was occluded from the ileocolic artery bifurcation. However, the 0.014-inch guidewire and 4-Fr catheter easily passed the occlusion lesion. Following them, the guiding sheath was placed close to the central side of the occlusion lesion. While moving the catheter, negative pressure was applied from the sheath to perform thrombus aspiration. This procedure was repeated several times, and small white materials and red thrombus were detected in the aspirated blood. After several aspiration, although some thrombus reduction was observed and the flow of the jejunal branch improved, thrombus still remained much and migrated to the central side. Judging that there was a limit to aspiration alone, we placed a 4 mm × 40 mm RIVAL (Bard, Covington, USA) into the occlusion area and PTA was carried out by inflating a balloon slowly. After PTA, the patient’s abdominal pain improved, and the thrombus grew smaller but remained. We injected urokinase solution (60 000 unit) into the artery, leaving the sheath and finishing the operation at once. Next day, further reduction of the thrombus was seen by contrast injection and additional PTA was performed using 4 mm × 40 mm Angiosculpt (PHILIPS, Amsterdam, Netherlands). After PTA, successful revascularization was achieved in almost all branches (Fig. 2). At this point, surgery was completed.

**Figure 3:**
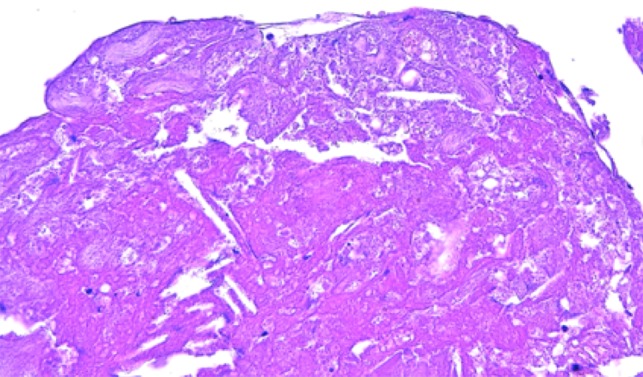
Pathological findings. Cholesterol clefts is observed.

Patient resumed oral feeding on the first day after operation, and TEVAR using Zenith alpha (Cook, Bloomington, USA) for descending aortic aneurysm was performed on the 28th day. The patient was discharged by Day 59 post-surgery with no complication, and there was no recurrence of abdominal symptoms or findings suggestive of intestinal ischemia throughout the course. The white material collected intraoperatively was found to be cholesterol crystals as a result of the pathological test (Fig. 3).

## DISCUSSION

We experienced SMA embolism after TEVAR. SMA embolism has a poor prognosis causing intestinal ischemia. It is important that diagnosis and treatment starts as soon as possible in order to improve prognosis. In addition to surgical treatment such as SMA bypass and surgical embolectomy, treatment by IVR has also been carried out in recent years, and there are reports that the result is relatively good [1–5]. Many previous studies used both thrombus aspiration and thrombolysis by urokinase or tissue type plasminogen activator [2–5]. According to this, we performed thrombus aspiration and thrombolysis to treat SMA embolism and we achieved a good result. We also performed PTA, because crush the hard thrombus and crimp it to the vessel wall. Of course, there was a risk of distal embolism, but it was performed to revascularize the trunk firmly.

Colonic ischemia after endovascular aortic repair (EVAR) occurs in 1-3% of patient, but intestinal ischemia after TEVAR seems to be a rare complication [6]. Zahn *et al.* reported the early and 1-year post-operative results of TEVAR for 191 patients with thoracic aortic disease. The results stated that among the reported patients, there were no reports of complications as SMA embolism or intestinal ischemia [7]. In our case, a thrombus in the aortic wall by the distal side of the stent graft was considered to be an embolus. Pathological findings also suggest that. TEVAR seemed to be the cause of this SMA embolism. Later, when TEVAR was performed on the descending thoracic aortic aneurysm, it was placed as to prevent any recurrence.

With the increased use of TEVAR, embolism related to TEVAR will also increase. Thrombus aspiration and PTA could be one effective option for such cases.
